# Supplementary material for: Band splitting with vanishing spin polarizations in noncentrosymmetric crystals
Source: Nat Commun. 2019 Nov 13;10:5144. doi: 10.1038/s41467-019-13197-z (PMC6854082; doi:10.1038/s41467-019-13197-z)
Supplement: Supplementary file 1 — Supplementary Information [file 41467_2019_13197_MOESM1_ESM.pdf]

Supplemental Material for:

Band splitting with vanishing spin polarizations in noncentrosymmetric crystals

By Liu et al.

Supplementary Note 1. Requirement of the non-SOC band structure for BSVSP

In Dresselhaus/Rashba spin splitting, inversion asymmetry lifts a spin-degenerate band without SOC into two single degenerate bands with SOC, accompanied by appearance of spin polarizations dependent on *k*, as shown in Fig. 1 of the main text. Here, we demonstrate that spin polarizations must be non-zero for an isolated spin-degenerate band when SOC occurs. Actually, the basis of these systems with SOC can be chosen as $|\phi_1\rangle = |\phi, \uparrow\rangle$ and $|\phi_2\rangle = |\phi, \downarrow\rangle$, where ϕ is the orbital part of the basis. The wave functions must be a linear combination of these two functions

$$|\psi_i\rangle = \nu_i|\phi_1\rangle + \mu_i|\phi_2\rangle, \quad (1)$$

where i = 1, 2 and ν_i, μ_i are coefficients satisfying the $|\nu_i|^2 + |\mu_i|^2 = 1$ condition. Note that the Bloch factor ($e^{i\vec{k}\cdot\vec{r}}$) is not included in the basis. The spin polarizations can be expressed as

$$\langle S_\alpha^i \rangle = \left\langle \psi_i \middle| \frac{\hbar}{2}\sigma_\alpha \middle| \psi_i \right\rangle \ (2)$$

where i = 1, 2, $\alpha = x, y, z$ and σ_α is Pauli matrix. If $\langle S_\alpha^i \rangle = 0$ for i = 1, 2 and $\alpha = x, y, z$, the solutions of υ_i, and μ_i must equal to zero. This fact means that there are no wave functions which display vanishing spin polarizations in this case. Therefore, there is no BSVSP in the systems where inversion asymmetry lifts a spin-degenerate band without SOC to two non-degenerate bands with SOC.

However, BSVSP may take place when the non-SOC eigenstates are at least two-fold degenerate (i.e., they belong to a 2D single-valued representation), as shown in Fig. 1 of the main text.

Supplementary Note 2. Group theory analysis for BSVSP

We focus on non-magnetic systems which respect the time-reversal symmetry. The existence of non-degenerate bands with SOC means that the little *double* point group at a given *k* point must have one-dimensional additional irreducible representations (IRs). The magnitude of spin polarizations of a Bloch state Ψ_k can be expressed as $\langle\psi_k|S_\alpha(k)|\psi_k\rangle$, where **S** is the spin operator and $\alpha = x, y, z$. For a non-degenerate state, its scalar product $\langle\psi_k|\psi_k\rangle$ belongs to the identical representation ($\tilde{\Gamma}_1$). The vanishing

spin polarizations of Ψ_k mean that $\langle\psi_k|S_\alpha(k)|\psi_k\rangle = 0$ when spin vectors S_x, S_y and S_z do not belong to the identical representation. BSVSP can not only occur in systems with symmorphic space groups, but also in systems with non-symmorphic space groups. In the following, we will discuss these two cases separately.

Symmorphic space groups. For crystals with symmorphic space groups, one can adopt the corresponding point group to analyze the symmetry properties of the electronic eigenstates. We start from finding which little point groups can display BSVSP at a given point **k**. The corresponding crystal point groups will be super-groups of the possible little point groups. First, the 11 centrosymmetric point groups (C_i, C_{2h}, D_{2h}, D_{3d}, S_6, D_{4h}, C_{4h}, D_{6h}, C_{6h}, O_h and T_h) should be excluded since they contain the inversion symmetry which enforces at least double Kramer degeneracy. Then, we also exclude the 11 point groups (C_{2v}, C_{4v}, C_{6v}, D_2, D_4, D_6, D_{3h}, D_{2d}, T, T_d and O) whose corresponding double point groups do not have one-dimensional additional IRs. Finally, only two (C_{3v} and D_3) of the 10 remaining point groups are non-pseudo-polar point groups. However, if the little point group associated with **k** is D_3, this k-point must be the Γ point. But all the eigenstates at Γ are at least two-fold degenerate since Γ point is a time-reversal invariant point. Therefore, the only possible little point group which might lead to BSVSP is C_{3v}. There are four non-centrosymmetric super-groups of C_{3v}, namely C_{3v}, D_{3h}, C_{6v} and T_d. In Table 1 of the main text, we list all the possible 4 symmorphic space groups which might display BSVSP. The corresponding high-symmetry lines where BSVSP takes place are also given.

Nonsymmorphic space groups. In the case of nonsymmorphic space groups, if the k-point at which BSVSP takes place is inside the Brillouin zone, we can adopt the method similar to that employed for the symmorphic case. This means that the little point group associated with the k-point inside the Brillouin zone must be the C_{3v} point group. If the k-point locates at the Brillouin zone boundary, the eigenstates can no longer be classified by the little point group. In this case, one has to check the nonsymmorphic space groups one by one to find out whether BSVSP is possible for the given space group. For a given nonsymmorphic space group, one first finds out the possible *k*-points on the Brillouin zone boundary where the corresponding little point

group has at least one 1D additional IRs. Then, among these *k*-points, one finds out the subset of the *k*-points whose corresponding little point group is a non-pseudo-polar point group. If the subset of the *k*-points is not null, BSVSP might take place for the given nonsymmorphic space group. To illustrate this process, we choose the noncentrosymmetric nonsymmorphic space group $Pmn2_1$ as an example. First, we find that there are 1D double-valued IRs of little space group along the symmetry line X → U. Then, the little point group of symmetry line X → U is found to be a non-pseudo-polar point group C_{2v}. Therefore, there might exist BSVSP in $Pmn2_1$ systems along the symmetry line X → U.

Supplementary Note 3. BSVSP in bulk GaAs derived with the *k·p* method

The $k{\cdot}p$ Hamiltonian of valence bands of bulk GaAs in the vicinity of Γ point is $H = H^+ + H^-$ as given by ref.[1] where H^+ is invariant with respect to the spatial inversion,

$$\begin{aligned} H^+ = \frac{\hbar^2}{m}[(\gamma_1 + \frac{5}{2}\gamma_2)\frac{1}{2}k^2 - \gamma_2(k_x^2 J_x^2 + k_y^2 J_y^2 + k_z^2 J_z^2) \\ -2\gamma_3(\{k_x, k_y\}\{J_x, J_y\} + \{k_y, k_z\}\{J_y, J_z\} + \{k_z, k_x\}\{J_z, J_x\})], \end{aligned} \tag{3}$$

while H^- breaks the spatial inversion symmetry,

$$H^- = -\frac{2C}{\sqrt{3}}[k_x\{J_x, V_x\} + k_y\{J_y, V_y\} + k_z\{J_z, V_z\}]. \tag{4}$$

Here, γ_1, γ_2, γ_3 and C are constants. J_x, J_y and J_z are 4×4 angular momentum matrices for a state of spin 3/2. k_x, k_y and k_z are the kinetic momentum operator. The symbol $\{a, b\}$ means the symmetrized product $\frac{1}{2}(ab + ba)$. The quantities V_x, V_y and V_z are given by: $V_x = J_y^2 - J_z^2$, $V_y = J_z^2 - J_x^2$, $V_z = J_x^2 - J_y^2$. Here, we focus on the band degeneracy and spin polarizations instead of the exact band energy. For simplicity, we set γ_1 = -3.6, γ_2 = 0.47, γ_3 = -0.7, $\hbar^2/m = 1$ and C = -0.1. The qualitative results do not depend on the exact parameters.

Along symmetry line Λ ($k_x = k_y = k_z = k$), there are two single degenerate eigenvalues $E_S^{\pm} = \pm\frac{\sqrt{6}}{5}k - \frac{6}{5}k^2$ whose corresponding eigenvectors are

$$|\psi_S^{\pm}\rangle = \mp\frac{\sqrt{2}}{4}(1-i)|\phi_1\rangle + \frac{\sqrt{3}}{6}(\mp\sqrt{2} - i)|\phi_2\rangle + \frac{\sqrt{3}}{12}[(-2 \mp \sqrt{2}) + (2 \mp \sqrt{2})i]|\phi_3\rangle + \frac{1}{2}|\phi_4\rangle, \tag{5}$$

and one double degenerate eigenvalue $E_d = -\frac{48}{5}k^2$ whose corresponding eigenvectors are

$$|\psi_d^1\rangle = \frac{1}{\sqrt{6}}[(-1-i)|\phi_1\rangle + \sqrt{3}i|\phi_2\rangle + |\phi_4\rangle],$$

$$|\psi_d^2\rangle = \frac{1}{\sqrt{6}}[\sqrt{3}i|\phi_1\rangle + (1-i)|\phi_2\rangle + |\phi_3\rangle]. \qquad (6)$$

Here, $|\phi_i\rangle$ (i = 1, 2, 3, 4) are the bases of the $k{\cdot}p$ Hamiltonian. These bases can be expressed in terms of atomic orbitals

$$|\phi_1\rangle = \left|j=\frac{3}{2}, M=\frac{3}{2}\right\rangle = -\frac{i}{\sqrt{2}}(|p_x\uparrow\rangle + i|p_y\uparrow\rangle),$$

$$|\phi_2\rangle = \left|j=\frac{3}{2}, M=\frac{1}{2}\right\rangle = -\frac{i}{\sqrt{6}}(|p_x\downarrow\rangle + i|p_y\downarrow\rangle - 2|p_z\uparrow\rangle),$$

$$|\phi_3\rangle = \left|j=\frac{3}{2}, M=-\frac{1}{2}\right\rangle = \frac{i}{\sqrt{6}}(|p_x\uparrow\rangle - i|p_y\uparrow\rangle + 2|p_z\downarrow\rangle),$$

$$|\phi_4\rangle = \left|j=\frac{3}{2}, M=-\frac{3}{2}\right\rangle = \frac{i}{\sqrt{2}}(|p_x\downarrow\rangle - i|p_y\downarrow\rangle). \qquad (7)$$

Through straightforward derivation, we can show that the magnitude of spin polarizations for single degenerate bands $\langle S_\alpha^i\rangle = \langle\psi_i|\frac{\hbar}{2}\sigma_\alpha|\psi_i\rangle = 0$, where i = 1, 2, α = x, y, z and σ_α is Pauli matrix, i.e., there is a BSVSP effect, as shown in Fig. 3(b).

To investigate the microscopic origin of the vanishing spin polarization along the symmetry line Λ, we calculate on-site energy differences between p orbitals (e.g., of the As^{3-} ion) with opposite spin orientations which can be regarded as the magnetic fields acting locally on the chosen orbitals. We find that

$$\langle p_x, s_x|H_\Lambda|p_x, s_x\rangle - \langle p_x, -s_x|H_\Lambda|p_x, -s_x\rangle = 0,$$

$$\langle p_x, s_y|H_\Lambda|p_x, s_y\rangle - \langle p_x, -s_y|H_\Lambda|p_x, -s_y\rangle = 2k,$$

$$\langle p_x, s_z|H_\Lambda|p_x, s_z\rangle - \langle p_x, -s_z|H_\Lambda|p_x, -s_z\rangle = -2k,$$

$$\langle p_y, s_x|H_\Lambda|p_y, s_x\rangle - \langle p_y, -s_x|H_\Lambda|p_y, -s_x\rangle = -2k,$$

$$\langle p_y, s_y|H_\Lambda|p_y, s_y\rangle - \langle p_y, -s_y|H_\Lambda|p_y, -s_y\rangle = 0,$$

$$\langle p_y, s_z|H_\Lambda|p_y, s_z\rangle - \langle p_y, -s_z|H_\Lambda|p_y, -s_z\rangle = 2k,$$

$$\langle p_z, s_x|H_\Lambda|p_z, s_x\rangle - \langle p_z, -s_x|H_\Lambda|p_z, -s_x\rangle = 2k,$$

$$\langle p_z, s_y|H_\Lambda|p_z, s_y\rangle - \langle p_z, -s_y|H_\Lambda|p_z, -s_y\rangle = -2k,$$

$$\langle p_z, s_z|H_\Lambda|p_z, s_z\rangle - \langle p_z, -s_z|H_\Lambda|p_z, -s_z\rangle = 0, \qquad (8)$$

where H_Λ is the Hamiltonian along the symmetry line Λ ($k_x = k_y = k_z = k$), and $|p_\alpha, \pm s_\beta\rangle$ represents the α orbital with the spin along the $\pm\beta$ direction. We see that the different p orbitals experience magnetic fields along different directions. Such effective magnetic fields are responsible for the band splitting. However, the net magnetic field on a given site vanishes; for instance, the magnetic fields along the z direction are $-2k, 2k, 0$ for p_x, p_y, p_z orbitals, respectively. The local magnetic moments associated with these orbitals will cancel with each other, resulting in a vanishing net spin polarization for the eigenstate and vanishing local spin polarizations for Ga and As atoms.

Supplementary Note 4. BSVSP in SnI_4

The space group of SnI_4 is $P\bar{4}3m$ whose corresponding point group T_d is the same as that of bulk GaAs. In Supplementary Fig. 1(a), we show the calculated SOC band structure and the x components of spin polarizations as a function of k in the vicinity of Γ point. The y and z components have the same behavior. Along symmetry line Λ (Γ → L), displaying C_{3v} symmetry as bulk GaAs with the rotational axis parallel to [111], the top non-degenerate bands ($\tilde{\Lambda}_4$ IR and $\tilde{\Lambda}_5$ IR, respectively) have vanishing spin polarizations, displaying the BSVSP behavior. For comparison, conventional Dresselhaus splitting with spin polarizations is presented along symmetry line K → Γ. The site symmetry of I atoms is C_{3v} with the rotational axis parallel to [111], [-111], [1-11] and [11-1] for I^1, I^2, I^3 and I^4, respectively. In Supplementary Fig. 1(b), we can see that spin polarizations projected on I^2, I^3 and I^4 atoms do not vanish because the intersection of site symmetry of these atoms and the little point group of symmetry line Λ is C_1 which is pseudo-polar point group. This situation is different from that in bulk GaAs.

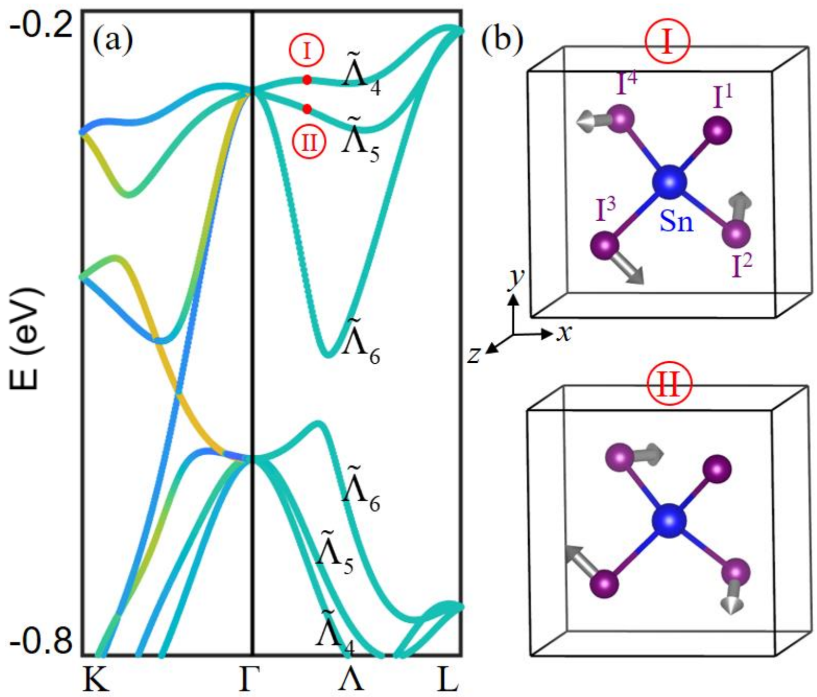

Supplementary Figure 1. BSVSP in SnI_4. (a) Band structure and the *x* components of spin polarizations of SnI_4 calculated by DFT. The color scheme is the same as that adopted in Fig. 1 of the main text. The coordinates of symmetry points K and L are (0.5,0.5,0) and (0.5,0.5,0.5), respectively. (b) Spin polarizations projected on Sn and I atoms for the wavefunctions marked by at the red points in (a). The blue and purple spheres are Sn and I atoms, respectively.

Supplementary Note 5. Spin polarizations of bulk GaAs with 2% in-plane compressive strain

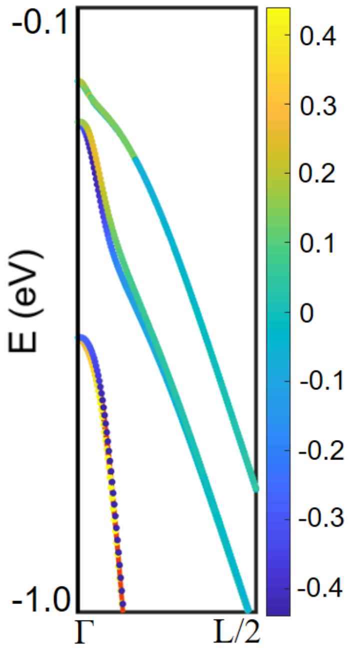

Supplementary Figure 2. The x component of spin polarizations of bulk GaAs with 2% in-plane compressive strain (i.e., decrease the lateral lattice constants) calculated by DFT. The y and z components also have non-zero spin polarizations and are not shown here. The color bar on the right represents the magnitude of spin polarization.

Supplementary Note 6. BSVSP in 2D ferroelectric SnTe derived from wave functions and *k·p* model

The band structure of 2D ferroelectric SnTe without SOC is shown in Supplementary Fig. 3. At high symmetry point X, the bands $\widetilde{\mathrm{X}}_1$ are double degenerate. We can define these two wave functions as $|\phi_1\rangle$ and $|\phi_2\rangle$. Based on wave functions $|\phi_1\rangle$ and $|\phi_2\rangle$, the representations of space group operators at symmetry point X can be written as

$$D(R_1) = \begin{pmatrix} 1 & 0 \\ 0 & 1 \end{pmatrix},\ D(R_2) = \begin{pmatrix} 0 & 1 \\ 1 & 0 \end{pmatrix},\ D(R_3) = \begin{pmatrix} 0 & -1 \\ 1 & 0 \end{pmatrix},\ D(R_4) = \begin{pmatrix} 1 & 0 \\ 0 & -1 \end{pmatrix}, \quad (9)$$

where $R_1 = \{E|0,0,0\}$ is the invariant operator, $R_2 = \{C_{2z}|1/2,0,1/2\}$ is twofold screw rotation C_{2z} which consists of π/2 rotation around the z axis followed by $a/2$ translation along the x axis and $c/2$ translation along the z axis, $R_3 = \{M_{xz}|1/2,0,1/2\}$ is reflection M_{xz} which consists of reflection about the $y = 0$ ($x = 0$) plane followed by $a/2$ translation along the x axis and $c/2$ translation along the z axis. $R_4 = \{M_{yz}|0,0,0\}$ is reflection M_{yz} which consists of reflection about the $x = 0$ plane.

The band structure of 2D ferroelectric SnTe with SOC is shown in Supplementary

Fig. 4. At high symmetry point X, the bands $\tilde{X}_1$ are split into two double degenerate bands $\tilde{X}_2$, $\tilde{X}_5$ and $\tilde{X}_3$, $\tilde{X}_4$. Note that these two double degenerate bands are protected by time inversion symmetry, respectively. By choosing wave functions $|\phi_{1,} \uparrow\rangle$, $|\phi_{1,} \downarrow\rangle$, $|\phi_{2,} \uparrow\rangle$ and $|\phi_{2,} \downarrow\rangle$ as bases, the representations of operators at symmetry point X can be written as

$$D(R_1) = \begin{pmatrix} 1 & 0 & 0 & 0 \\ 0 & 1 & 0 & 0 \\ 0 & 0 & 1 & 0 \\ 0 & 0 & 0 & 1 \end{pmatrix}, \; D(R_2) = \begin{pmatrix} 0 & 0 & -i & 0 \\ 0 & 0 & 0 & i \\ -i & 0 & 0 & 0 \\ 0 & i & 0 & 0 \end{pmatrix},$$

$$D(R_3) = \begin{pmatrix} 0 & 0 & 0 & 1 \\ 0 & 0 & -1 & 0 \\ 0 & -1 & 0 & 0 \\ 1 & 0 & 0 & 0 \end{pmatrix}, \; D(R_4) = \begin{pmatrix} 0 & -i & 0 & 0 \\ -i & 0 & 0 & 0 \\ 0 & 0 & 0 & i \\ 0 & 0 & i & 0 \end{pmatrix},$$

$$D(\bar{R}_1) = \begin{pmatrix} -1 & 0 & 0 & 0 \\ 0 & -1 & 0 & 0 \\ 0 & 0 & -1 & 0 \\ 0 & 0 & 0 & -1 \end{pmatrix}, \; D(\bar{R}_2) = \begin{pmatrix} 0 & 0 & i & 0 \\ 0 & 0 & 0 & -i \\ i & 0 & 0 & 0 \\ 0 & -i & 0 & 0 \end{pmatrix},$$

$$D(\bar{R}_3) = \begin{pmatrix} 0 & 0 & 0 & -1 \\ 0 & 0 & 1 & 0 \\ 0 & 1 & 0 & 0 \\ -1 & 0 & 0 & 0 \end{pmatrix}, \; D(\bar{R}_4) = \begin{pmatrix} 0 & i & 0 & 0 \\ i & 0 & 0 & 0 \\ 0 & 0 & 0 & -i \\ 0 & 0 & -i & 0 \end{pmatrix}. \quad (10)$$

These reducible representations can be reduced into IRs,

$$D(R) = \tilde{X}_2(R) \oplus \tilde{X}_3(R) \oplus \tilde{X}_4(R) \oplus \tilde{X}_5(R), \quad (11)$$

where $R = R_1, R_2, R_3, R_4, \bar{R}_1, \bar{R}_2, \bar{R}_3, \bar{R}_4$ and the overline means that the operator is additional.

To obtain the eigenvectors related to IRs, we define the project operators:

$$P_{\tilde{X}_2} = \frac{1}{8}(R_1 + iR_2 - R_3 + iR_4 - \bar{R}_1 - i\bar{R}_2 + \bar{R}_3 - i\bar{R}_4),$$

$$P_{\tilde{X}_3} = \frac{1}{8}(R_1 - iR_2 + R_3 + iR_4 - \bar{R}_1 + i\bar{R}_2 - \bar{R}_3 - i\bar{R}_4),$$

$$P_{\tilde{X}_4} = \frac{1}{8}(R_1 + iR_2 + R_3 - iR_4 - \bar{R}_1 - i\bar{R}_2 - \bar{R}_3 + i\bar{R}_4),$$

$$P_{\tilde{X}_5} = \frac{1}{8}(R_1 - iR_2 - R_3 - iR_4 - \bar{R}_1 + i\bar{R}_2 + \bar{R}_3 + i\bar{R}_4). \quad (12)$$

Choosing a function $|F\rangle = |\phi_{1,} \uparrow\rangle + |\phi_{1,} \downarrow\rangle + |\phi_{2,} \uparrow\rangle + |\phi_{2,} \downarrow\rangle$ and applying the project operators, we obtain

$$|\psi_{\tilde{X}_2}\rangle = P_{\tilde{X}_2}|F\rangle = \frac{1}{2}(|\phi_{1,} \uparrow\rangle + |\phi_{1,} \downarrow\rangle + |\phi_{2,} \uparrow\rangle - |\phi_{2,} \downarrow\rangle),$$

$$|\psi_{\tilde{X}_3}\rangle = P_{\tilde{X}_3}|F\rangle = \frac{1}{2}(|\phi_{1,} \uparrow\rangle + |\phi_{1,} \downarrow\rangle - |\phi_{2,} \uparrow\rangle + |\phi_{2,} \downarrow\rangle),$$

$$|\psi_{\tilde{X}_4}\rangle = P_{\tilde{X}_4}|F\rangle = \frac{1}{2}(|\phi_{1,} \uparrow\rangle - |\phi_{1,} \downarrow\rangle + |\phi_{2,} \uparrow\rangle + |\phi_{2,} \downarrow\rangle),$$

$$|\psi_{\tilde{X}_5}\rangle = P_{\tilde{X}_5}|F\rangle = \frac{1}{2}(-|\phi_{1,} \uparrow\rangle + |\phi_{1,} \downarrow\rangle + |\phi_{2,} \uparrow\rangle + |\phi_{2,} \downarrow\rangle), \quad (13)$$

where $|\psi_{\tilde{X}_i}\rangle$ (i = 2, 3,4 ,5) is the eigenvector of IR $\tilde{X}_i$ and they are mutually orthogonal. Through straightforward derivation, we can show that the magnitude of spin polarizations for these wave functions $\langle S_\alpha^i\rangle = \left\langle \psi_{\tilde{X}_i} \middle| \frac{\hbar}{2}\sigma_\alpha \middle| \psi_{\tilde{X}_i} \right\rangle = 0$, where i = 2, 3, 4, 5, $\alpha = x, y, z$ and σ_α is Pauli matrix.

From symmetry point X to symmetry line Σ, the spatial symmetry does not change. Therefore, we can also choose $|\psi_{\tilde{X}_i}\rangle$ (i = 2, 3,4 ,5) as the basis of IR $\tilde{\Sigma}_i$. Note that the bands $\tilde{\Sigma}_2$, $\tilde{\Sigma}_5$ and $\tilde{\Sigma}_3$, $\tilde{\Sigma}_4$ are not degenerate because time-reversal symmetry is broken. As above, the magnitude of spin polarizations equal to zero, i.e. there is BSVSP.

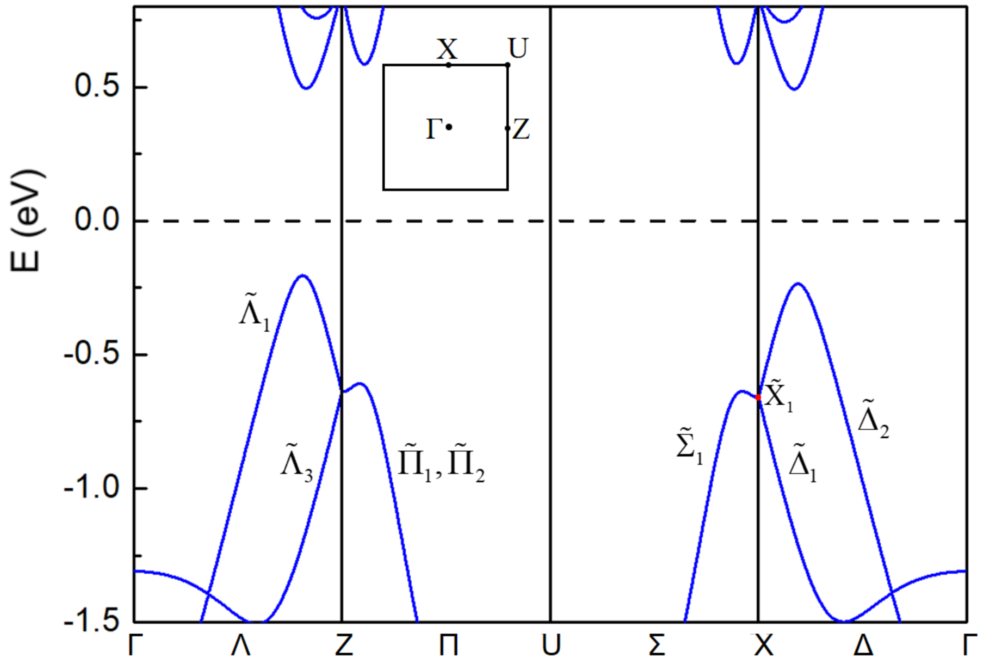

Supplementary Figure 3. Band structure of 2D ferroelectric SnTe without SOC. The IR $\tilde{\Sigma}_1$ and $\tilde{X}_1$ are two dimensional. The other IRs are one dimensional.[2]

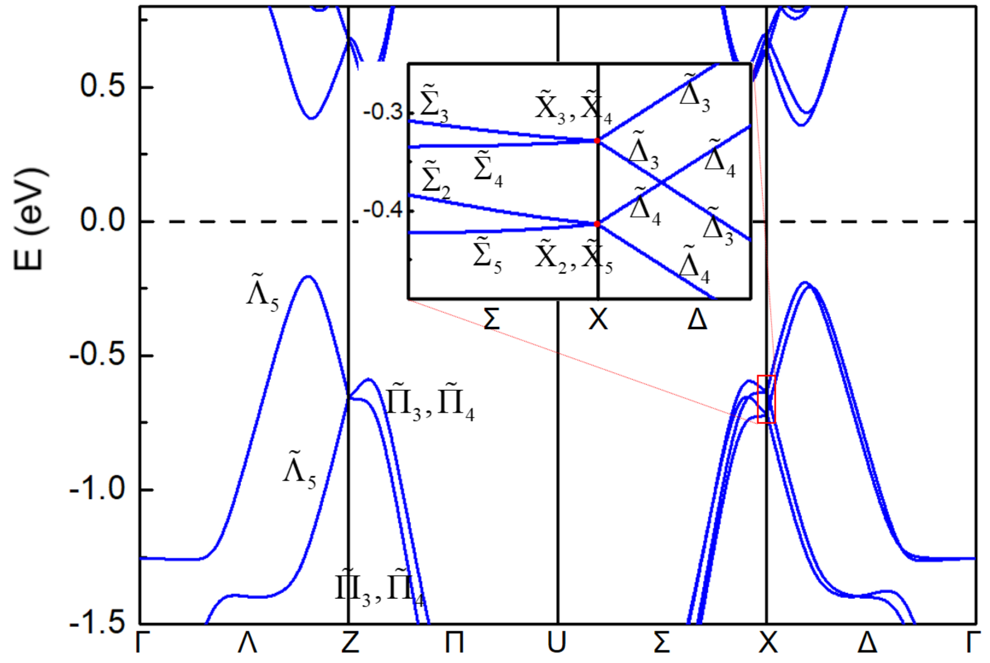

Supplementary Figure 4. Band structure of 2D ferroelectric SnTe with SOC. The IR $\tilde{\Lambda}_5$ is two dimensional. The other IRs are one dimensional.[2] For the symmetry line along Z → U, the bands are two-fold degenerate due to the non-symmorphic symmetry. This is rather uncommon, since the Rashba effect usually leads to band splitting in the plane that is perpendicular to the polar direction (z axis here). In fact, the bands along Z → U line can be described by the $k{\cdot}p$ model $H = \alpha_R k_x \sigma_y \tau_z$, i.e., as two copies of Rashba-split bands with opposite spin polarization along the y axis. This is analogous to the hidden spin polarizations predicted in centrosymmetric systems, where the vanishing of the net spin polarization is due to the non-symmorphic symmetry.

The band structure around point X can be further described by using the $k{\cdot}p$ perturbation theory, starting from the unperturbed wave functions $|\phi_1\rangle$ and $|\phi_2\rangle$ defining the basis of the $\tilde{\mathrm{X}}_1$ IR. The perturbed Hamiltonian reads $H = H_1 + H_2 + H_3$, where

$$H_1 = \frac{\hbar}{m} k \cdot p,\ H_2 = \frac{\hbar}{4m^2c^2}(\nabla U \times p) \cdot \sigma,\ H_3 = \frac{\hbar^2}{4m^2c^2}(\nabla U \times k) \cdot \sigma. \quad (14)$$

The matrix elements of the perturbed Hamiltonians in terms of the unperturbed wave functions can be determined by solving the systems of linear equations defined as:

$$\langle \phi_i | O_\beta | \phi_j \rangle = \frac{1}{h} \sum_R \sum_{i',j',\beta'} D^*{}_{i'i}(R)\, {}^{O}D_{\beta'\beta}(R) D_{j'j}(R) \langle \phi_{i'} | O_{\beta'} | \phi_{j'} \rangle, \quad (15)$$

where h is the order of the group, $D(R)$ are the matrices of the symmetry operation in the $\tilde{X}_1$ IR while ${}^{O}D(R)$ are the symmetry operations of a given operator $\boldsymbol{O}$. Using the character table for the polar vectors ($\boldsymbol{p}$ and ∇U) and the axial vector ($\nabla U \times p$) in Supplementary Table 1, and enforcing the time-reversal symmetry, one gets the following $k{\cdot}p$ Hamiltonian:

$$H_{\tilde{X}_1} = \begin{pmatrix} -\alpha_B(k_z\sigma_x - k_x\sigma_z) + \beta_R k_x \sigma_y & i\Delta\sigma_y - i\alpha_R k_x \sigma_0 \\ -i\Delta\sigma_y + i\alpha_R k_x\sigma_0 & \alpha_B(k_z\sigma_x - k_x\sigma_z) + \beta_R k_x \sigma_y \end{pmatrix}, \quad (16)$$

where:

$$\alpha_B = \langle \phi_1 | (\nabla U)_y | \phi_1 \rangle,\ \beta_R = \langle \phi_1 | (\nabla U)_z | \phi_1 \rangle,$$

$$i\Delta = \langle \phi_1 | (\nabla U \times p)_y | \phi_2 \rangle,\ i\alpha_R = \langle \phi_1 | p_x | \phi_2 \rangle.$$

As expected, its eigenstates along the symmetry line Σ (k_x = 0) coincide with those obtained through the projection operators, showing BSVSP.

Supplementary Table 1. Character table of the symmetry operations of C_{2v} point group for polar (**P**) and axial (**A**) vectors.

	R_1	R_2	R_3	R_4
P_x (A_y)	1	-1	1	-1
P_y (A_x)	1	-1	-1	1
P_z	1	1	1	1
A_z	1	1	-1	-1

To investigate the microscopic origin of BSVSP in the SnTe thin-film, we calculate the magnetic field acted on the orbitals. From the DFT calculation, we find that the two-fold degenerate states at the X-point in the non-SOC case can be described as $|\phi_1\rangle = \frac{1}{\sqrt{2}}(|\mathrm{Sn1}, s\rangle - |\mathrm{Sn2}, s\rangle)$ and $|\phi_2\rangle = \frac{1}{\sqrt{2}}(-|\mathrm{Sn3}, s\rangle + |\mathrm{Sn4}, s\rangle)$ where Sn1, Sn2, Sn3 and Sn4 atoms are defined in Supplementary Fig. 5 and s means the 5s orbital of the Sn atom (Note that the Te contributions are neglected for simplicity). Along the symmetry line Σ (k_x = 0), the Hamiltonian can be written as $H_\Sigma = -\Delta\tau_y\sigma_y - \alpha_B k_z \tau_z \sigma_x$. The on-site energy differences between s orbitals of Sn atoms with different spin orientations

(regarded as the magnetic fields acted locally on the s orbitals of Sn atoms) are calculated to be

$$\langle \mathrm{Sn1}, +s_x | H_\Sigma | \mathrm{Sn1}, +s_x \rangle - \langle \mathrm{Sn1}, -s_x | H_\Sigma | \mathrm{Sn1}, -s_x \rangle = \alpha_B k_z,$$
$$\langle \mathrm{Sn2}, +s_x | H_\Sigma | \mathrm{Sn}_2, +s_x \rangle - \langle \mathrm{Sn2}, -s_x | H_\Sigma | \mathrm{Sn}_2, -s_x \rangle = \alpha_B k_z,$$
$$\langle \mathrm{Sn3}, +s_x | H_\Sigma | \mathrm{Sn3}, +s_x \rangle - \langle \mathrm{Sn3}, -s_x | H_\Sigma | \mathrm{Sn}_3, -s_x \rangle = -\alpha_B k_z,$$
$$\langle \mathrm{Sn4}, +s_x | H_\Sigma | \mathrm{Sn}_4, +s_x \rangle - \langle \mathrm{Sn4}, -s_x | H_\Sigma | \mathrm{Sn}_4, -s_x \rangle = -\alpha_B k_z,$$
$$\langle \mathrm{Sn1}, +s_y | H_\Sigma | \mathrm{Sn}_1, +s_y \rangle - \langle \mathrm{Sn1}, -s_y | H_\Sigma | \mathrm{Sn}_1, -s_y \rangle = 0,$$
$$\langle \mathrm{Sn2}, +s_y | H_\Sigma | \mathrm{Sn}_2, +s_y \rangle - \langle \mathrm{Sn2}, -s_y | H_\Sigma | \mathrm{Sn}_2, -s_y \rangle = 0,$$
$$\langle \mathrm{Sn3}, +s_y | H_\Sigma | \mathrm{Sn3}, +s_y \rangle - \langle \mathrm{Sn3}, -s_y | H_\Sigma | \mathrm{Sn}_3, -s_y \rangle = 0,$$
$$\langle \mathrm{Sn4}, +s_y | H | \mathrm{Sn}_4, +s_y \rangle - \langle \mathrm{Sn4}, -s_y | H_\Sigma | \mathrm{Sn}_4, -s_y \rangle = 0,$$
$$\langle \mathrm{Sn1}, +s_z | H_\Sigma | \mathrm{Sn}_1, +s_z \rangle - \langle \mathrm{Sn1}, -s_z | H_\Sigma | \mathrm{Sn}_1, -s_z \rangle = 0,$$
$$\langle \mathrm{Sn2}, +s_z | H_\Sigma | \mathrm{Sn}_2, +s_z \rangle - \langle \mathrm{Sn2}, -s_z | H | \mathrm{Sn}_2, -s_z \rangle = 0,$$
$$\langle \mathrm{Sn3}, +s_z | H_\Sigma | \mathrm{Sn3}, +s_z \rangle - \langle \mathrm{Sn3}, -s_z | H_\Sigma | \mathrm{Sn}_3, -s_z \rangle = 0,$$
$$\langle \mathrm{Sn4}, +s_z | H_\Sigma | \mathrm{Sn}_4, +s_z \rangle - \langle \mathrm{Sn4}, -s_z | H_\Sigma | \mathrm{Sn}_4, -s_z \rangle = 0. \qquad (17)$$

where $\pm s_\gamma$ ($\gamma = x, y, z$) denotes the spin direction and the orbital label s is omitted. We can see that Sn1 and Sn2 atoms on the upper layer experience the opposite magnetic fields along the x-axis as the Sn3 and Sn4 atoms on the lower layer, as shown in Supplementary Fig. 5. This staggered magnetic field induces the band splitting, while the net spin polarization is zero since the spin arrangement is AFM-like [see Fig. 4(b)]. This also implies that it is possible that BSVSP might occur for s orbitals.

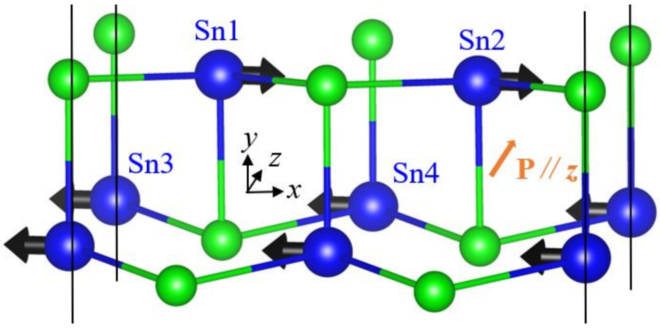

Supplementary Figure 5. The magnetic fields acted locally on Sn atoms. Sn1, Sn2, Sn3, and Sn4 are denoted.

Supplementary Note 7. Spin polarizations of the SnTe thin film induced by an external electric field

Our DFT calculations show that an external electric field along the *y*-direction can induce a net spin polarization along the *x*-direction in the SnTe thin film for the symmetry line Σ (see Fig. 5 of the main text). When the external electric field is reversed to be along the –*y* direction, the net spin polarization is also reversed (see Supplementary Fig. 6).

We now discuss the microscopic mechanism of the electric field effect. When an external electric field is applied, there is an additional contribution H_4 to the total Hamiltonian *H*: $H = H_1 + H_2 + H_3 + H_4$ with $H_4 = -E_y y$ (E_y represents the amplitude of the electric field along the *y*-axis). Subsequently, there is an additional term in the $k{\cdot}p$ Hamiltonian besides the zero-field Hamiltonian $H_{\tilde{X}_1}$:

$$H^E_{\tilde{X}_1} = H_{\tilde{X}_1} + \begin{pmatrix} -\alpha_E\sigma_0 & 0 \\ 0 & \alpha_E\sigma_0 \end{pmatrix}, \quad (18)$$

where $\alpha_E = -E_y\langle\phi_1|y|\phi_1\rangle$. Solving this Hamiltonian, we find that

$$\langle\psi_1|S_x|\psi_1\rangle = \frac{\alpha_E^2+\alpha_E\sqrt{\alpha_E^2+\Delta^2}}{\Delta^2} \approx \frac{\alpha_E^2}{\Delta^2} + \frac{\alpha_E}{\Delta},$$

$$\langle\psi_2|S_x|\psi_2\rangle = \frac{-\alpha_E^2-\alpha_E\sqrt{\alpha_E^2+\Delta^2}}{\Delta^2} \approx -\frac{\alpha_E^2}{\Delta^2} - \frac{\alpha_E}{\Delta},$$

$$\langle\psi_3|S_x|\psi_3\rangle = \frac{\alpha_E^2-\alpha_E\sqrt{\alpha_E^2+\Delta^2}}{\Delta^2} \approx \frac{\alpha_E^2}{\Delta^2} - \frac{\alpha_E}{\Delta},$$

$$\langle\psi_4|S_x|\psi_4\rangle = \frac{-\alpha_E^2+\alpha_E\sqrt{\alpha_E^2+\Delta^2}}{\Delta^2} \approx -\frac{\alpha_E^2}{\Delta^2} + \frac{\alpha_E}{\Delta}, \quad (19)$$

where ψ_i are the wavefunctions of the eigenstates. Therefore, the new Hamiltonian predicts that there is a non-zero spin polarization along the symmetry line Σ (k_x = 0), in agreement with the DFT result shown in Fig. 5(a) of the main text. When the electric field is reversed (i.e., E_y becomes $-E_y$), the spin polarization is also reversed, in agreement with the DFT result shown in Supplementary Fig. 6.

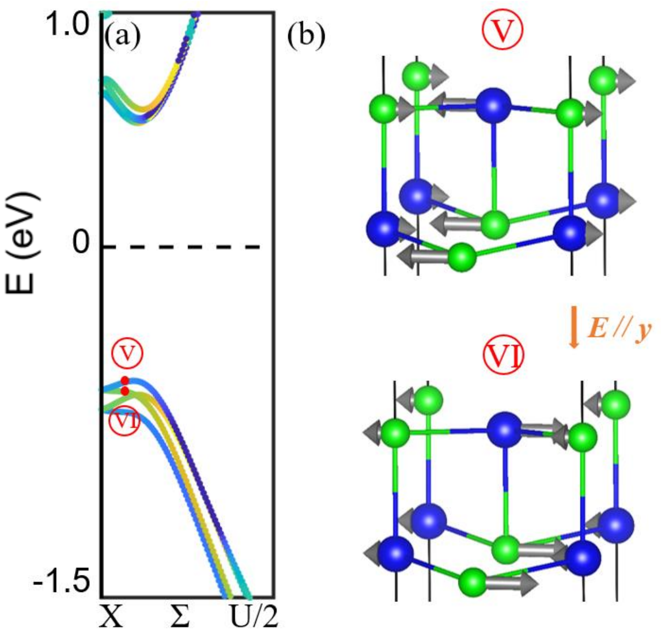

Supplementary Figure 6. Linear magnetoelectric coupling in reciprocal space in 2D SnTe thin film. (a) The *x* components of spin polarizations when a 0.1 V/Å external electric field antiparallel to *y* axis is added. The vanishing *y* and *z* components are not shown. The color bar of the spin polarization is the same as that in Fig. 5 of the main text. (b) Spin polarizations projected on Sn and Te atoms for the wavefunctions at the red points V and VI in (a).

Supplementary References

1 Pidgeon, C. R. & Groves, S. H. Inversion-Asymmetry and Warping-Induced Interband Magneto-Optical Transitions in InSb. *Phys. Rev.* **186**, 824-833 (1969).

2 Elcoro, L. *et al.* Double crystallographic groups and their representations on the Bilbao Crystallographic Server. *J. Appl. Crystallogr.* **50**, 1457-1477 (2017).
